# Translational precision medicine: an industry perspective

**DOI:** 10.1186/s12967-021-02910-6

**Published:** 2021-06-05

**Authors:** Dominik Hartl, Valeria de Luca, Anna Kostikova, Jason Laramie, Scott Kennedy, Enrico Ferrero, Richard Siegel, Martin Fink, Sohail Ahmed, John Millholland, Alexander Schuhmacher, Markus Hinder, Luca Piali, Adrian Roth

**Affiliations:** 1 Novartis Institutes for BioMedical Research, Basel, Switzerland; 2 Department of Pediatrics I, University of Tübingen, Tübingen, Germany; 3 Novartis Institutes for BioMedical Research, Cambridge, MA USA; 4 Basel, Switzerland; 5 Novartis Precision Medicine, Cambridge, MA USA; 6 Reutlingen University, Reutlingen, Germany; 7 Roche Innovation Center Basel, Basel, Switzerland

**Keywords:** Translational medicine, Precision medicine, Drug development, Biomarkers, Multi-omics, Modeling, Artificial intelligence, Pharmaceutical industry, Digital biomarkers, Companion diagnostics

## Abstract

In the era of precision medicine, digital technologies and artificial intelligence, drug discovery and development face unprecedented opportunities for product and business model innovation, fundamentally changing the traditional approach of how drugs are discovered, developed and marketed. Critical to this transformation is the adoption of new technologies in the drug development process, catalyzing the transition from serendipity-driven to data-driven medicine. This paradigm shift comes with a need for both translation and precision, leading to a modern *Translational Precision Medicine* approach to drug discovery and development. Key components of *Translational Precision Medicine* are multi-omics profiling, digital biomarkers, model-based data integration, artificial intelligence*,* biomarker-guided trial designs and patient-centric companion diagnostics. In this review, we summarize and critically discuss the potential and challenges of *Translational Precision Medicine* from a cross-industry perspective.

## Background

Traditionally, drug development in large pharmaceutical companies is regarded as a conservative and risk-averse discipline with highly regulated processes and slow adaptation to external innovation. However, in a rapidly evolving healthcare ecosystem, new technologies and innovative concepts of how to leverage them are needed to accelerate clinical trials, lower attrition rates, mitigate research and development (R&D)-related risks and overall improve pharmaceutical R&D productivity [1, 2]. Critical for future R&D success is the combination of transformative therapeutic concepts and drug targets with first-in-class potential, tailored digital technologies and patient-centric drug development, linked to a broader paradigm shift from *one-size-fits-all* medicine towards precision medicine (*the right medicine, for the right patient, at the right dose, at the right time*) [3, 4]. While precision medicine is an appealing concept, there are several core challenges for implementation from bench to the bedside, as discussed previously [5–7].

One of the major bottlenecks for drug development is *translation* [8], particularly at the interface of drug discovery and early clinical development, referred to as the *Translational Gap* [8–10]. To close this gap and foster translational science, the National Institutes of Health (NIH) has established the *National Center for Advancing Translational Sciences*, a core hub to drive and integrate innovative translational activities across academia, industry and non-profit organizations [11]. Translational medicine as defined by the *European Society for Translational Medicine* [12] integrates several R&D tools to bridge the translational gap and guide early drug development. Since translational medicine and precision medicine approaches in drug development are overlapping and intertwined, we use here the term *Translational Precision Medicine* to refer to this emerging discipline.

**Fig. 1:**
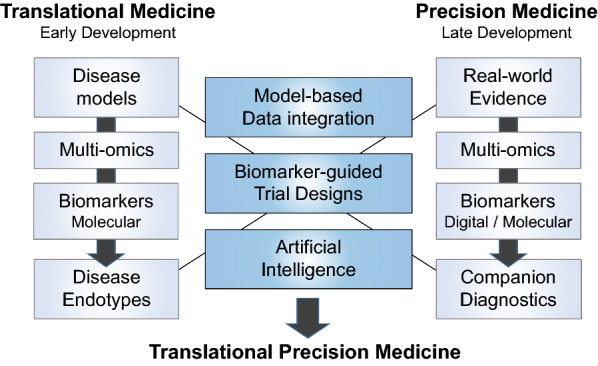
Interface position of *Translational Precision Medicine* in bridging translational medicine (early development) and precision medicine (late development). Disease models, multi-omics and molecular biomarkers are used to define disease endotypes. Real-world evidence, multi-omics, biomarkers (digital and molecular) and companion diagnostics are instrumental for the implementation of precision medicine. Model-based data integration, biomarker-guided trial designs and artificial intelligence are key data-driven tools for the integration of mechanism-centric translational medicine and patient-centric precision medicine

The *Translational Precision Medicine* concept integrates core components from both translational medicine (mechanism-based early drug development) and precision medicine (patient-centric late drug development) into an end-to-end biomarker-guided drug development cycle. Critical success factors for *Translational Precision Medicine* are (i) the translation of mechanisms from research to early clinical development (*forward translation/bench-to-bedside*), (ii) the back-translation from late clinical development insights to drug discovery (*reverse translation*/*back-translation*/*bedside-to-bench*) [13], (iii) data-driven mechanism-indication pairing [14], (iv) the translation of omics signatures into clinically-relevant biomarkers and endotypes [15] and (v) the development of patient-tailored companion diagnostics and precision medicines [3]. Here we focus on the following key components of *Translational Precision Medicine* (Fig. 1):

- Multi-omics profiling
- Biomarker-guided trial designs
- Model-based data integration
- Artificial Intelligence (AI)
- Digital biomarkers
- Patient engagement.

## Multi-omics profiling

Clinical data can be classified as phenotypic (such as demographics, physiologic assessments, disease scorings, imaging, health questionnaires, digital patient assessments) or molecular (such as genomics, transcriptomics, proteomics, metabolomics). Capturing comprehensive phenotypic data associated with a certain disease can be referred to as *phenotyping* (or *phenomics*), which is the traditional and most common approach to classify diseases irrespective of the biological origins of disease. Utilizing datasets to define disease subtypes at the molecular level can be referred to as *endotyping*, as exemplified in respiratory medicine [16, 17] or oncology [18]. The National Academy of Sciences of the USA campaigned for a new, molecularly-informed taxonomy to define diseases based on molecular endotypes rather than traditional clinical symptoms [19]. However, endotyping requires deep pathophysiological disease insights and large molecular datasets to be successful. Within the last two decades, high-throughput omics technologies have provided the basis for endotyping and data-driven medicine [20, 21]. With the rapid advances of sequencing technology, genetics has revolutionized our understanding of monogenic diseases, such as cystic fibrosis [22] or mutation-driven cancers [23], but most human diseases are polygenic and consequently more complex to dissect. To approach these diseases at the genetic level, polygenic risk scores hold promise to predict genetic predisposition to disease or therapeutics [24], particularly if combined with electronic health records (EHRs) [25]. Translational genomics aims to combine genetic and clinical data as a foundation for precision medicine approaches [7]. Besides genetics, particularly proteomics [26, 27] are gaining momentum for clinical biomarkers and drug development [28]. Unlike mass spectrometry, next-generation proteomic detection principles, such as aptamer-based technologies [29], typically require lower amounts of material (down to 1ul/sample) and can be more readily applied to large patient cohorts to identify causal proteins as candidates for therapeutic targeting [28, 30–33]. Compared to more established omics layers, such as transcriptomics, proteomics offer the benefit of measuring protein levels directly, thus facilitating the translation to the clinic where protein biomarkers are most commonly used.

Beyond single omics technologies, *multi-omics* profiling platforms are emerging, including *genomics, epigenomics, transcriptomics, proteomics, lipidomics, metabolomics, microbiomics* and others [34, 35]. Multi-omics profiling integrates several biological layers, allowing researchers to fully appreciate the interplay between genetics, gene regulation and proteins, and to obtain a more complete picture of the molecular patterns underpinning complex diseases. Thus, multi-omics are well positioned to enable the identification of key disease nodes where multiple layers converge, maximizing the chances to identify novel drug targets, endotypes or biomarkers. Networks offer an effective way to integrate and visualize the output of multi-omics analyses, particularly when the evidence does not converge at the level of a single gene, but within a biological pathway [36], and network propagation approaches can be applied to leverage network topology for the identification of key nodes [37]. Multi-omics are further essential for *N*-of-1 trials [38], for understanding drug-drug interactions and for the design of therapeutic drug combinations [39–41]. Despite this potential, there are several caveats and limitations of multi-omics when applied to clinical drug development:

- Omics technologies assess large numbers of genes/proteins, often in a semi-quantitative manner, and are highly sensitive to pre-analytical processes [42] such as batch effects [43]. Consequently, to build robust cases for clinical adoption, it is essential to include appropriate controls in the experimental design and to validate top hits by orthogonal quantitative methods [43, 44].
- Merging different multi-omics datasets [45] into a single data repository poses challenges to data transfer, integration and harmonization given different data formats and data fragmentation. Moreover, analyzing large complex datasets, such as single-cell multi-omics [46], increases the chance for false positives and necessitates appropriate data processing, normalization and analysis with appropriate statistical methods [44, 47].
- Clinical trial feasibility [15, 48], especially for multi-center and tissue-derived omics, remains a challenge. Restricting the number of well-selected clinical sites, strict standard operating procedures (SOPs), cross-site controls and qualified analytical core facilities are essential for robust data generation. Well-curated biobanks [49] are further pivotal to link multi-omics data to disease characteristics and clinical trial outcomes. Alignment on human biosample accessibility, FAIR data principles [50] and dissemination policies are also key for successful multi-omics collaboration networks.

## Biomarker-guided trial designs

Biomarkers are defined by the Biomarkers Definitions Working Group of the NIH/FDA, as “*a characteristic that is objectively measured and evaluated as an indicator of normal biological processes, pathogenic processes, or pharmacologic responses to a therapeutic intervention*” [51]. In drug development, biomarkers are broadly used to inform on target engagement, pathway activation, pharmacokinetic/pharmacodynamic (PK/PD) modeling and dosing rationales, diagnosis/patient selection, disease stratification, prognosis and prediction as well as monitoring disease, safety and treatment efficacy. Biomarkers are classified into molecular, cellular, physiological, imaging and digital modalities. As clinical trial endpoints, biomarkers provide the advantage of being quantitative and objective measures of (patho)biology in contrast to physician-based assessments which tend to be subjective and variable. Biomarkers are key to translate PD responses across species and to bridge the translational gap in early drug development [9, 10, 52], particularly for multifactorial systemic diseases [53] such as systemic immune-mediated diseases. From a drug development perspective, the longitudinal analysis of the AstraZeneca small molecule portfolio (five-dimensional (*5R*) framework) demonstrated that the inclusion of biomarkers into early drug development (Ph2 studies) was associated with active or successful projects in contrast to comparable projects without biomarkers [2].

The development of a new biomarker is a complex, multistep and iterative process, including biomarker discovery (often based on omics data), pre-analytical validation, assessing different biofluids (best proximal to the disease), analytical validation and finally clinical validation and utility [48]. For each new drug target and disease indication, several biomarker modalities and candidates are usually explored to narrow-down on the drug target- and indication-relevant ones, as discussed here for autoimmune diseases such as rheumatoid arthritis [54]. For biomarker use in clinical trials it is critical to define the *context-of-use* (CoU) [15, 55, 56]. CoU range from diagnostic, safety monitoring, PD response, to predictive and prognostic biomarker applications. For a detailed list of biomarker CoU, the reader is referred to the FDA-NIH biomarker working group and its related online resource *BEST (Biomarkers, Endpoints and other Tools)* [51]. Prognostic and predictive CoU are essential for clinical drug development: *prognostic* biomarkers at baseline are indicative of disease outcome independent from interventions (important to identify patients on high-risk for trial enrichment), whereas *predictive* biomarkers at baseline are indicative of response to a specific treatment (response prediction).

There are two basic paths how to integrate biomarkers in drug development: (1) within the context of a *specific drug development program* or (2) the official *FDA biomarker qualification program* (BQP). The *specific drug development program* path is the most common strategy pursued in pharmaceutical industry, where the drug developer / sponsor includes selected biomarkers in clinical trials, mainly for internal decision-making and is responsible for all aspects of the biomarker development. The FDA BQP is required to qualify biomarkers as general drug development tools [57] to make them applicable for multiple drug development programs and to qualify them as regulatory drug approval tools, which is a formal and lengthy process usually involving consortia composed of multiple academic and industry partners. In the FDA BQP, the candidate biomarker is qualified for a pre-specified CoU. The FDA provides an updated online list of BQP-qualified biomarkers [58]. Of note, qualifying a biomarker for a CoU via the BQP or qualifying a specific test measuring a biomarker are two different and independent approaches. For biomarker test/assay qualification, e.g. to develop a companion diagnostic assay (see below), pre-analytical and assay performance characteristics are key. Beyond the US/FDA, other regional/national biomarker guidances and regulatory frameworks, such as guidances from European Medicines Agency (EMA), Asian-Pacific (APAC) regulators, National Medical Products Administration (NMPA) and/or Pharmaceuticals and Medical Devices Agency (PDMA), have to be taken into account for biomarker qualification and clinical implementation.

In general, the level of impact that biomarkers can have depends on three key factors: (i) the validation and qualification status of the biomarker, (ii) the CoU and (iii) the scientific evidence linking the biomarker with the CoU. Biomarkers in clinical trials are mostly used as exploratory endpoints to explore new mechanistic hypothesis and inform internal decision making. If biomarkers are deemed more impactful and clinically relevant, biomarkers are used as secondary or primary clinical endpoints. Typical examples here are physiological biomarkers like blood pressure, clinically-established protein biomarkers such as C-reactive protein, or imaging readouts. If there is convincing evidence from independent epidemiological studies and clinical trials that biomarkers correlate closely with clinical outcome assessments, biomarkers can be considered to substitute for a clinical endpoint as *surrogate endpoints*, which have a major relevance for diseases with outcomes that take a long time to capture using traditional clinical endpoints. Examples here are systolic blood pressure for occurrence of stroke or low-density lipoprotein cholesterol levels for occurrence of heart attacks. For implementation of biomarkers in pharmaceutical industry trials, several drug development aspects have to be further taken into account, including informed consent/data protection considerations, clinical trial logistics/feasibility, impact on clinical decision-making and cost-effectiveness [48, 59–61].

To actively guide clinical trial flows, *biomarker-guided trial designs* are the method of choice [62, 63], which are particularly useful for novel clinical trial designs using master protocols (basket, umbrella and adaptive platform trials) [64]. For a comprehensive overview on biomarker-guided trial designs, the reader is referred to the *BiGTeD online resource* [65]. For biomarker-guided trial designs, biomarkers should be analyzed in Clinical Laboratory Improvement Amendments (CLIA) certified (for US) or equivalent (non-US) labs. The two most commonly applied biomarker-guided trial designs are *stratification* [66] and *enrichment* [67]. Biomarker-based stratification, or stratified randomization, means that biomarkers are measured in all patients prior to randomization and are used to proportionally/equally balance treatment vs placebo arms with respect to biomarker status. Biomarker-stratified designs have the advantage that patients are not excluded if they are biomarker-negative. The next more stringent level of biomarker trial design is enrichment. For that design, inclusion of the individual patient into the clinical trial is depending on a defined biomarker assessment. Quality requirements for biomarkers and analytical labs are higher when using this approach, as protocol-defined treatment decisions depend directly on the biomarker. Enrichment designs can be especially useful for situations when it is not ethically justified to treat biomarker-negative patients based on biomarker-response and/or biomarker-safety relationships, such as CYP metabolism. The recent FDA guidance on enrichment [68] should be taken into account that recommends smart enrichment, adaptive enrichment and the inclusion of a biomarker-negative population in at least one trial before NDA/MAA submission (with defined exceptions). Besides stratification and enrichment designs, other more complex biomarker-guided trials designs are summarized as *biomarker-strategy designs* [65]. All biomarker-guided trial designs can be implemented in non-adaptive or adaptive settings. The latter provides more flexibility for the trial, yet is also more challenging to implement. Apart from interventional biomarker-guided trial designs, non-interventional (observational) biomarker-guided trial designs using master protocols have been proposed recently in the oncology field (*Master observational trials*) [69].

**Fig. 2:**
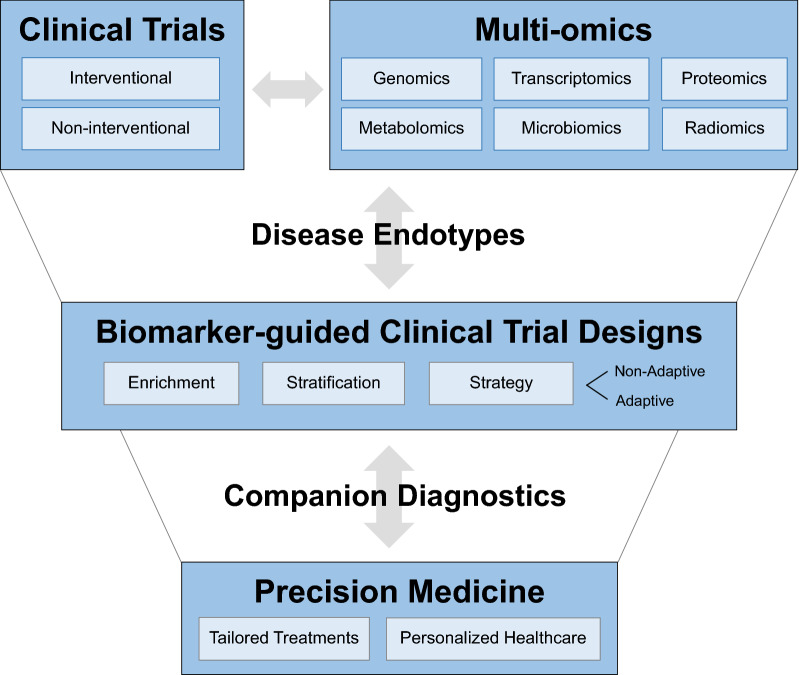
Flow of clinical trials (interventional or non-interventional) integrating multi-omics approaches to identify disease endotypes, which enables biomarker-guided trials designs (adaptive or non-adaptive) and paves the way towards precision medicine approaches (tailoring treatments for personalised healthcare)

Biomarker-guided trial designs ultimately pave the way towards precision medicine, i.e. tailoring drug development to specific patient characteristics [3, 70]. In 2015, the US government launched a *Precision Medicine Initiative* [71, 72]. Precision medicine focuses on individual rather than average responses to therapy and led to the concept of *N*-of-1 trials [38], ideally based on longitudinal multi-omics data. While precision medicine approaches are already widely implemented in oncology and rare genetic diseases, other therapeutic areas have just begun to tailor drug development based on these principles [3]. Biomarkers can enable precision medicine through the development of *companion diagnostics* [73–75], mainly established in oncology [76]. Companion diagnostics are classified as in vitro diagnostic (IVD) medical devices (IVD class I, II or III) and are typically co-developed with the drug to increase response rates by lowering the *numbers-needed-to-treat* and/or to spare patients exposure to drugs that have a high probability to fail or even cause harm. The development path for an exploratory biomarker to a full IVD companion diagnostic is complex, requires at-risk investments and should start early in drug development in close alignment with health authorities [74, 75, 77, 78]. Companion diagnostics should be broadly available and accessible to relevant healthcare professionals for clinical routine use. A list of cleared or approved companion diagnostic devices is provided by FDA [79]. Companion diagnostics [80] are strictly regulated by health authorities/FDA [81] and are differentiated from complementary diagnostics [82, 83] as they are essential for treatment decisions, whereas complementary diagnostics just support treatment decisions. As biopsy-derived tissue is often challenging to obtain from non-oncology patients, “liquid biopsies” (derived from peripheral blood/serum/plasma) are a major domain of companion diagnostics, yet assay performance characteristics, such as sensitivity and specificity, are key for success in that area. Figure 2 illustrates the flow from multi-omics-based endotyping, over biomarker-guided trial designs to companion diagnostics-based precision medicine approaches.

To increase the benefit-risk ratio of drug candidates, safety aspects are increasingly becoming an integral part of biomarker-guided precision medicine approaches. For example, the observation that patients receiving checkpoint inhibitor therapy experiencing immune-related adverse events also exhibit an improved treatment response was recently shown to be related to a polygenic risk score [84]. Furthermore, for the first time, a polygenic risk score could be established for the prediction of drug-induced liver injury, a common and very difficult to predict adverse event in the clinic [85].

When viewed in combination, biomarker-guided trial designs provide ideal tools to catalyze the transition from an empirical and *physician-centric* to a data-driven and *patient-centric* precision medicine approach [70, 86, 87]. However, technical (companion diagnostic assay development), clinical (complex biomarker-guided trial designs, master protocols) and regulatory (requirements for companion diagnostics as medical devices) hurdles have to be tackled, particularly in non-oncology indications [83, 88].

## Model-based data integration

Given the small size and low number of samples per subject in pre-clinical experiments as well as early clinical trials, analyzing and leveraging biomarker data in translational medicine remains challenging. One way for improving the statistical power for detecting signals is to use longitudinal (i.e. time-dependent) model-based data integration. Mathematical models are used to describe the time course of PK and PD/biomarker results for better understanding of the pharmacology and to predict future experimental outcomes [89]. Already in the 1930s, mathematical equations were used to describe PK data [90], but the start of more extensive model-based approaches took off with the development of computers and was brought into drug-development in the 1970s–80s. The need has been highest for compounds with a small therapeutic window where dose-individualization was needed. This was especially challenging for compounds with a substantial delay between the exposure and the biomarker PD response. Therefore, the initial approaches for describing inter-subject variability (population “PopPKPD” models) were developed in anesthesiology [91] and for anticoagulants (e.g. warfarin [92]).

Population models most often use simplified model structures to model the observed data (i.e. mainly measured in plasma/blood or *ex-vivo*). When considering chemotherapies, like methotrexate, questions arose about the tumor-relevant tissue distribution of the compound [93]. Thus, a second class of models, i.e., physiologically-based, were developed to describe the whole Liberation, Absorption, Distribution, Metabolism, Excretion (LADME) processes in more detail for small molecules, where these processes are heavily dependent on their physico-chemical properties [94]. This approach is used to predict drug-drug interactions, but also for scaling from animals to humans in the translational medicine realm – alternatively to PopPK models.

Scaling PK parameters across species is mainly based on allometric scaling, which describes the weight-dependence of physiological aspects (volumes, metabolic rates, clearance, etc.) within and between species using power functions with fixed exponents. This works well for PK parameters [93], yet can be challenging for biomarkers due to inter-species differences of pathway expression, production rates or whole physiological networks. These approaches have gained more traction by integrating systems biology and quantitative systems pharmacology [95]. A third concept is to apply physical and biological assumptions, such as monotonous exposure–response, continuity/smoothness of underlying signals or allometric scaling. These models can be used for high-dimensional data (like multi-omics) to reduce noise when multiple sampling time points are available per individual.

Finally, the FDA has established a new framework for model-informed drug development [96]: “*FDA is conducting a Model-Informed Drug Development (MIDD) Pilot Program to facilitate the development and application of exposure-based, biological, and statistical models derived from preclinical and clinical data sources, referred to as MIDD approaches. MIDD approaches use a variety of quantitative methods to help balance the risks and benefits of drug products in development. When successfully applied, MIDD approaches can improve clinical trial efficiency, increase the probability of regulatory success, and optimize drug dosing/therapeutic individualization in the absence of dedicated trials.*” This MIDD pilot program is based on joint discussions between the pharmaceutical industry and the European health authorities/EMA, which led to a paper in 2016 on good modeling practices [97]. The latter includes the concept of the learning-and-confirming circle of modeling as well as drug development, where model-based predictions inform the next study design, e.g., predictions of PK and efficacy or safety biomarkers from animal data into first-in-man studies. The acceptance or even push from health authorities for MIDD approaches [98] indicates the high value of model-based data integration.

## Artificial Intelligence

The amount of data generated and collected in pharmaceutical R&D is increasing at an unprecedented pace. Combined with improvements in information processing and more powerful hardware, machine learning, deep learning and AI in general are positioned to disrupt drug discovery and development towards an algorithm-based R&D [99]. Deep learning has already revolutionized several industries, particularly in the area of image analysis and recognition, while its impact in biomedical R&D remains to be fully embraced [100]. High-dimensional multi-omics datasets derived from large longitudinal clinical studies provide an ideal ground for the application of machine learning [101] and AI [99]. Examples with an impact on drug discovery and development include: target identification [102–104], biomarker discovery [105, 106] and patient endotyping [107, 108]. Machine learning and deep learning algorithms are also powerful analytical methods when applied to digital biomarkers data, allowing to transform longitudinal, multi-modal and complex raw data from sensors and connected digital devices into endpoints and clinically-relevant measures [109, 110].

Given the rigidity of traditional serendipity- and forward translation-based drug development frameworks, shifting to a new mindset embracing the use of AI for the discovery and development of drugs is a critical success factor for *Translational Precision Medicine* [111]. A comprehensive cross-industry analysis recently mapped out AI-related activities across major pharmaceutical companies [112], coming to the conclusion that, compared with leading technology companies (e.g., Microsoft, Google), most pharmaceutical organizations are still in an *early mature* phase of using AI in R&D. However, an increasing number of healthcare companies have now started their digital journey, building up AI competencies and data literacy across many areas of R&D [112]. For example, Johnson & Johnson and Novartis have started to commercialize AI-based products and services in healthcare. Medical AI application focused so far mainly on the diagnosis of disease conditions based on EHRs, digital pathology and biomarkers [113, 114]. To go beyond and fully leverage AI technologies for clinical drug development, it is essential to optimize and validate AI algorithms for use in clinical trials and outcome prediction. AI-powered approaches have the potential to enable precision medicine, particularly in chronic disease conditions, by dissecting complex high-dimensional patient datasets and tailoring drug development [115]. While traditionally regulatory authorities might not have been perceived as enthusiastic about advanced AI models in biomedical R&D, the landscape is evolving rapidly, exemplified by recent developments in the AI-based medical device space [116, 117] and the recent FDA pilot program *Innovative Science and Technology Approaches for New Drugs* (ISTAND) that incentivizes the use of AI-based algorithms to evaluate patients, develop novel endpoints, or inform study designs. Moving forward, it will be critical that pharmaceutical organizations continue to constructively engage early on with regulatory authorities on innovative ways to design and assess clinical trials, including a more widespread use of AI technologies in drug development.

Overall, the impact of AI in drug discovery and clinical development will largely depend on the underlying data, and its intrinsic limitations. AI-based analysis of both multi-omics as well as EHRs depends critically on the quality and quantity of the provided molecular and clinical datasets, key limitations and challenges that need to be overcome in the future.

The near future will show whether and how these emerging AI algorithms will help scientists to (i) identify novel targets or new indications for existing drugs, (ii) uncover latent factors that can inform on disease pathogenesis or drug response, (iii) discover predictive biomarkers enabling patient stratification strategies that can optimize clinical trial designs, and (iv) ultimately impact the drug development value chain. For more detailed overviews of AI in drug discovery and development, we refer the reader to dedicated reviews in this field [87, 101, 111, 118].

## Digital biomarkers

The recent evolution of sensor technologies and the widespread use of smartphones and other connected digital products are enabling the comprehensive collection and analysis of health-related data [119–121]. Progress in algorithms and analytical methodologies to transform sensor data into clinical insights have facilitated the rapid development of digital biomarkers [122, 123]. Digital biomarkers are defined as physiological and behavioral measures collected via digital devices (such as portables, wearables, implantables and digestables) that characterize, influence or predict health-related outcomes [124, 125]. Digital biomarkers offer several potential advantages compared to traditional clinical assessments. Objective data can be collected in real-life settings, in a quantitative and unbiased way and on a frequent or continuous basis, resulting in increased statistical power, and enhanced sensitivity and specificity [122, 126]. In clinical trials, these characteristics allow for lower sample size, fewer study visits, shorter study duration and real-time feedback for early decision-making [120, 122, 126, 127]. Longitudinal digital patient data can be leveraged to advance precision/personalized medicine approaches. Furthermore, the use of digital biomarkers in drug development enables patient centricity, integration of real-world evidence, reduced patient burden of trial participation, increased inclusivity in patient enrollment [121], decentralized trials [128] and better product differentiation [129]. Despite being a promising new technology, a major requirement and challenge for digital biomarkers is to ensure protection of relevant sensitive patient data in the whole process.

Successful examples of digital biomarkers are in the field of neurodegenerative diseases, where traditional clinical outcome measures are sparse, highly variable and rater-dependent [130]. Smartphone-based measurements have been developed and deployed in clinical trials to monitor signs of Parkinson’s disease [131, 132]; while features from inertial measurement unit features have been recently benchmarked to predict Parkinson’s disease severity [133]. Susceptibility/risk biomarkers from computerized cognitive testing are in use to classify adults at high risk of late-onset of Alzheimer’s disease [134, 135]. Clinically relevant gait parameters from inertial wearable sensor were identified to assess gait impairment in Huntington disease [136].

While the number of studies involving digital technologies is growing and extending to more technologies, biomarker categories and therapeutic areas [119, 121], the use of digital biomarkers as clinical endpoints is today still in an early research phase due to several layers of complexity. Digital biomarker products are usually the result of the combination of multiple individual hardware (sensors) and software (operating systems and algorithms) components [123]. Hence is it vital to thoroughly verify technology and analytical solutions and clinically validate digital biomarkers in the desired cohorts and context of use, prior to their adoption as clinical endpoints [123, 127, 137]. The majority of current efforts still have an engineering focus and address algorithm development and sensor performance [120]. So far, very limited solutions are undergoing clinical validation.

Transforming digital device data into validated clinical endpoints is a lengthy process, which involves the collaboration of multiple disciplines, from engineering, machine learning, data science, clinical research and regulatory interactions. An open validation framework based on transparency, metadata standards, external validation and data sharing is necessary to harmonize approaches and evaluate and improve digital biomarkers in clinical settings [123]. Recently, multiple concrete efforts have emerged and are shaping and accelerating the development of validated digital biomarkers: (i) guidelines from the *Clinical Trials Transformation Initiative* (CTTI) [138], the *Digital Medicine Society* [139] and the EMA [140]; (ii) pragmatic fit-for-purpose validation frameworks [137, 141]; (iii) open-source platforms such as the *Digital Biomarker Discovery Pipeline* [142]; (iv) open benchmarking challenges [143]; (v) and several Innovative Medicine Initiative (IMI) programs, such as MOBILISE-D [144], IDEA-FAST [145] and RADAR-AD [146].

Future opportunities for digital biomarkers towards patient-centric precision medicine are (i) algorithms based on longitudinal/real-time composite biomarkers from multiple connected technologies and contextual information in real-world settings [123], (ii) integration of molecular/multi-omics and digital biomarkers, and (iii) digital phenotyping for patient stratification [147–149].

## Patient engagement

Since the AIDS pandemic in the 1980s, the way industry interacts with patients has changed fundamentally, from passive recipients to active contributors along the whole drug development value chain. This has been particularly evident in the last decade, where most pharmaceutical companies have started patient engagement groups to actively listen to the patient voice [150, 151]. Industry has finally realized that patient engagement is not an additional burden, but can improve and actually accelerate drug development. Similarly, health authorities increasingly incorporate the patient voice into their regulatory guidance [152]. For example, The FDA’s *Patient-Focused Drug Development* initiative led to the guidance for industry on how to best identify what is important to patients. In Europe, the EMA formed its *Patients’ and Consumers’ Working Party*. Engagement with patients, their caregivers, patient experts and patient advocacy groups have been shown to yield benefit for both patients and the industry [153, 154]. Increased patient involvement in the process ensures that industry focuses on the real medical needs, that study protocols are patient-centric and that new treatments become available faster. Conversely, industry benefits from a more robust identification of patients` needs, faster conduct of clinical trials, a quicker path to market and overall higher credibility and sustainability [155]. Patients not only have increased their involvement with industry, but likewise with regulatory authorities and sit in governing bodies. Major milestones are the foundation of the *International Alliance of Patient’s Organizations*, the *Patient-Centered Outcomes Research Institute* [156] and the *Patient-focused Medicines Development* [157] among several other patient-centric initiatives. In fact, the impact of patient engagement throughout the healthcare ecosystem is driving change at various levels: becoming a credible source for patients themselves, improving access and care, driving R&D and advocating for policy changes in collaboration with governments.

Traditionally, patient engagement has been mainly considered once a new drug is already on the market. The majority of decisions about the molecule and its clinical development path, including unmet medical needs, have then already been taken by the company. Studies, however, demonstrated that the early integration of the patient perspective, particularly in preclinical research and early development, has the biggest impact on value creation for patients, business and society [158]. As preclinical research is a discipline that usually does not collaborate directly with patients, a change of mindset to include the patient voice already at this stage can be challenging, yet represents the clear future towards *patient-led research* [159]. A recent paper identified key challenges of implementing patient engagement in preclinical research and provided possible solutions to overcome current barriers [160]. In interviews with patient groups, industry and academia conducted by the CTTI, patient representatives identified engagement with research partners as having particularly great benefit. Patient-led organizations are keen to learn more about their diseases and are highly interested to collaborate in research projects and willing to provide their data (anonymized and under strict data protection policies) for research and clinical development [161]. A key recommendation for industry is to engage the patient voice as early as possible from the beginning of the R&D program to improve trial design and clinical execution [162].

Personalised healthcare (PHC), precision medicine and stratified medicine have been used interchangeably to describe the concept of tailoring treatment to patients based on their individual pathology. With the rise of new diagnostic and data-driven approaches that deepen our understanding of the molecular basis of disease, this centuries-old dream has come closer to reality. Nowadays, the awareness of the potential of PHC is also emerging in the patient community and its meaning goes far beyond precision medicine. PHC comprises everything that allows to tailor treatment and medical care by combining conventional clinical datasets, molecular signatures (such as genetics), environment, lifestyle and personal needs. Some of the key innovations in that area include digital healthcare solutions with technologies connecting digital patient information/EHRs with wearable devices, mobile Apps, telehealth and digital assistants using AI [163], see also the respective chapters above. A major requirement and challenge for that field is to protect relevant sensitive patient data and patient rights in that whole process. Patients, caregivers and healthcare providers are acknowledging the utility and advancement offered by these approaches in key domains, such as patient education, accurate diagnosis, patient outcomes, quality of life, disease prevention and health care value [164], more recently underscored by different initiatives, such as the EU Health Data Space race [165] or the US Precision Medicine initiative [166]. The overall goal in all of this is to make healthcare decisions jointly together with the patient as an integrated R&D partner.

## Conclusions

*Translational Precision Medicine* comes with a paradigm shift from a *one-size-fits-all* to a biomarker-guided patient-centric medicine. Key success factors for adoption of this principle in pharmaceutical drug development include the combination of forward and reverse translation, the classification of disease conditions as multi-omics-defined endotypes, the integration of AI- and algorithm-based R&D concepts, the implementation of digital biomarkers as clinical endpoints and the development of companion diagnostics. The rise of data-driven and algorithm-based R&D necessitates the establishment of a new mindset of how data mining and AI tools can be used effectively to discover and develop new drugs [111]. The near future will show whether and how these emerging AI-based digital tools will reveal new targets, pathogenic disease signatures, optimize clinical trial designs and overall impact drug development across pharmaceutical industries. Convergence of patient-centric real-world evidence (RWE) tools, EHRs, multi-omics profiling, digital biomarkers and AI-based data analysis will pave the way towards biomarker-enabled algorithm-based precision R&D.

## Outlook

The *Translational Precision Medicine* evolution comes with distinct challenges: (i) *multi-omics* data are mainly useful to drug discovery and development if they reveal new drug targets or biomarker signatures that correlate with disease outcome and/or treatment response [61]; (ii) multi-omics-based patient and disease stratification requires accurate diagnoses and detailed clinical annotations/EHRs; (iii) *digital biomarkers* as clinical endpoints provide objective and quantitative measures yet still require broader clinical use and health authority acceptance; (iv) *biomarker-guided trial designs* and *precision medicine* approaches are already widely implemented in oncology and rare diseases, while other non-oncology areas have just started to pursue these concepts and (v) *precision medicine*/*companion diagnostics* approaches come with substantial development costs and reimbursement hurdles. One important question is how these novel technologies and assessments are perceived by patients, as acceptance and adherence to clinical read-outs is key for patient trial recruitment and long-term engagement. Novel patient-centric interaction approaches are currently implemented to engage patients more pro-actively in R&D, RWE networks and clinical trials. New cloud-based data systems and platforms for interactions with regulatory agencies [167], for sharing datasets between industry and academia, for public–private partnerships or for managing cross-industry partnerships and multidisciplinary initiatives like the *Information Exchange and Data Transformation* (INFORMED) initiative of the FDA [168] will further shape the way towards data-driven medicine.

The COVID-19 era substantially disrupted the traditional pharmaceutical R&D approach at several layers [169–172]: (i) virtual, data-based, data-sharing (including open repositories such as bioRxiv and medRxiv) and collaborative research and drug discovery/development concepts are getting higher traction; (ii) large longitudinal datasets collected from COVID-19 patients are systematically analyzed and offer great potential for multi-omics [173–175] and AI-based analyses [169, 176–178], supporting diagnosis, basic disease understanding, endotyping, image analysis, drug target identification and drug repurposing [169, 179]; (iii) clinical trials are accelerated, decentralized and increasingly include digital endpoints, biosensors, home nursing, patient-centric sampling and remote clinical trial recruitment and monitoring strategies [171, 180], accompanied by a FDA guidance on conduct of clinical crials during COVID-19 [181]. In combination, these emerging concepts rapidly and successfully implemented during the COVID-19 outbreak hold promise to make drug discovery and development more efficient and less burdensome to patients also beyond the pandemic era.

Emerging therapeutic modalities, including CAR T-cells [182], gene therapy [183, 184], induced protein degradation [185] or mRNA-based principles [186, 187], and patient-derived organoids for ex vivo drug response testing to guide personalized treatments [188] add further levels of complexity to biomarker-guided translational precision. Finally, the core future challenge for *Translational Precision Medicine* as for drug development overall remains how to leverage and embrace new molecular and digital technologies in a way that is feasible for larger clinical trials, accepted by regulators and, most importantly, by patients.

## Data Availability

Not applicable.

## Abbreviations

AI: Artificial Intelligence
BEST: Biomarkers, Endpoints and other Tools
BQP: Biomarker qualification program
CLIA: Clinical Laboratory Improvement Amendments
CoU: Context-of-use
CTTI: Clinical Trials Transformation Initiative
EHR: Electronic health records
EMA: European Medicines Agency
FDA: U.S. Food and Drug Administration
IVD: In vitro diagnostic
LADME: Liberation, Absorption, Distribution, Metabolism, Excretion
MIDD: Model-Informed Drug Development
NIH: National Institutes of Health
PD: Pharmacodynamic
PHC: Personalised healthcare
PK: Pharmacokinetic
R&D: Research and development
RWE: Real-world evidence
SOP: Standard operating procedures
